# Deep sequence learning with multi-task supervision for scalable population health monitoring

**DOI:** 10.3389/fpubh.2026.1837769

**Published:** 2026-06-29

**Authors:** Imran Ashraf, Inzamam Mashood Nasir, Muhammad Awais, Wided Bouchelligua, Sahar Mansour, Essa Alyounis, Majed Nawaz

**Affiliations:** 1 Computer Engineering Lab, Quantum and Computer Engineering Department, EEMCS, TU Delft, Delft, Netherlands; 2 Human-Environment-Technology (HET) Systems Centre, Mykolas Romeris University, Vilnius, Lithuania; 3 Department of Computer Science, College of Computer, Qassim University, Buraydah, Saudi Arabia; 4 Applied College, Imam Mohammad Ibn Saud Islamic University (IMSIU), Riyadh, Saudi Arabia; 5 Department of Radiological Sciences, College of Health and Rehabilitation Sciences, Princess Nourah bint Abdulrahman University, Riyadh, Saudi Arabia; 6 Department of Health Information Management and Technology, College of Applied Medical Science, King Faisal University, Al Ahsa, Saudi Arabia; 7 Department of Computer Science, College of Science, Northern Border University, Arar, Saudi Arabia

**Keywords:** deep learning, early disease, health analytics, longitudinal healthcare data, multi-task sequence modeling, population health surveillance, risk prediction

## Abstract

Models designed for artificial intelligence-driven population health monitoring must be able to integrate multiple types of information over time, be heterogeneous in the data they consider, and be deployed at scale. This research proposes an integrated multitask framework for predicting population disease incidence and mortality risk, based on deep learning and using both longitudinal biobank and National Health Survey data in a scalable manner. The framework will learn shared temporal representations from clinical lab-generated/demographically defined survey variables by applying a strict prospective evaluation approach across the framework. On UK Biobank, the proposed model achieves an AUROC of 0.842 and a *C*-index of 0.791, outperforming strong baselines such as Proformer having AUROC 0.829 and UKB-MDRMF having AUROC 0.821. On NHANES, the model achieves an AUROC of 0.861 and a *C*-index of 0.808, exceeding those of commonly used boosted tree and survival-based approaches. Temporal robustness analysis shows controlled performance degradation across extended horizons, with UK Biobank AUROC decreasing from 0.872 at 1 year to 0.819 at 10 years and NHANES AUROC decreasing from 0.886 at 1 year to 0.836 at 5 years. Subgroup evaluation demonstrates stable performance across age, sex, and clinical risk strata, while scalability analysis confirms near-linear training growth and stable inference latency below three milliseconds per individual at biobank scale.

## 1 Introduction

Sustainable public health systems increasingly rely on timely population-level intelligence to anticipate health risks, allocate limited resources, and guide preventive interventions. The growing availability of large-scale healthcare data, including longitudinal biobank records, electronic health records, national health surveys, and linked environmental and behavioral indicators, has created new opportunities for data-driven population health surveillance. At the same time, this data growth has exposed fundamental limitations of traditional epidemiological and statistical approaches, which often struggle to scale across heterogeneous data sources, long temporal horizons, and multiple disease outcomes (1).

Recent years have therefore witnessed a shift toward artificial intelligence and deep learning methods for population health analysis. Unlike classical risk scoring systems, modern learning-based approaches can model complex non-linear interactions across demographic, clinical, and biological variables, enabling more accurate prediction of disease incidence, progression, and mortality at scale (2). These abilities are relevant to sustainable healthcare systems, where early risk identification and stratification can reduce unnecessary clinical utilization, enhance targeted prevention efforts, and increase the long-term efficiency of the system (3).

Conventional methods for healthcare risk modeling often use static features that depict just the baseline or final observations from longitudinal patient data. Although still clinically relevant for specific endpoints, these methods miss important temporal relationships, irregular observation intervals, and the changing dynamics of health that are present in most population monitoring systems. In the context of extensive public health monitoring systems, health care data are collected asynchronously over a long period, with substantial variation in follow-up intervals, temporal sparsity, and the types of data collected for different individuals. This means the temporal aspect of the data must be addressed before modeling. The proposed method addresses this issue by ensuring temporal data validity during cohort formation, defining surveillance intervals, aligning data, and learning data sequences. This method design enables the framework to identify longitudinal disease progression while preserving the causal structure, which is necessary for estimating population-level risks.

Population cohorts, such as biobanks (usually nationally based), comprise large numbers of individuals and are essential to advancing data-driven surveillance. Longitudinal cohorts allow researchers to model disease trajectory over several decades, which in turn affords early detection of chronic conditions and patterns of multi-morbidity that place ongoing demands on the healthcare system (4). Recent advances in longitudinal healthcare modeling have increasingly explored both deep learning and conventional machine learning approaches for population-level risk prediction. Deep sequence architectures and representation learning frameworks excel at modeling temporal interdependencies and sampling patterns prevalent in the myriad of multimodal trajectories found in large-scale healthcare datasets. Recent studies have shown that, for organized healthcare datasets, traditional boosting-based and survival-based models remain competitive with deep learning models. This emphasizes the importance of utilizing temporality-aware modeling techniques. Deep sequence learning enables modeling heterogeneous attributes with long-term temporal relationships and the interdependencies across multiple dimensions in large health datasets (5).

The merging methodologies of longitudinal ML and population health surveillance have recently pivoted away from isolation and predictive accuracy toward temporal robustness, subgroup stability, and reliable calibration and scalability within the bounds of real-world deployment. Recent findings suggest that both transformer-based and longitudinally optimized ML frameworks can yield substantial improvements when the temporal structure of a cohort is preserved during both construction and evaluation. Maintaining prospective temporal consistency, these findings stimulate the design of these frameworks and incorporate a surveillance approach to longitudinal observations. At the same time, population health surveillance also benefits from representative survey samples, such as NHANES. NHANES is a population-based survey that includes laboratory testing, clinical measurements, socioeconomic status, and many other items needed for providing an overall picture of population health trends and differences in health across various subgroups of the population (6). Machine learning and deep learning methods on NHANES have demonstrated the potential for risk stratification, disease prevalence estimation, and mortality prediction, supporting scalable public health planning and evaluation (7, 8).

The majority of existing research has focused on narrowly defined prediction tasks or relied on a single dataset; therefore, these findings may not be applicable to other population-wide health surveillance goals (9). In addition, heterogeneous data from health care sources may contain missing information, be susceptible to sampling bias, and vary over time, making it difficult to deploy learning-based models in real-world public health workflows (10–12). To overcome these barriers, scalable modeling strategies will be necessary that can integrate multiple data types while remaining sufficiently robust and interpretable.

Most traditional prediction frameworks involve static snapshots of patient data aggregated at specific time points. The framework proposed here, however, accounts for population health as a dynamic system that continually evolves over time. This difference becomes significant in the context of scalable health surveillance systems, where the incidence of disease and the risk of death are related to clinical variables, the time intervals between health events, the persistence, recurrence, and temporal interactions among health events. This surveillance framework, through temporally organized latent structures, can model long-term health transitions and capture delayed health dependencies better than traditional regression, survival, and cross-sectional methods based on machine learning. The interconnected representation of population health is maximally enhanced by integrating multi-task learning, providing a cohesive, unified predictive framework for correlated population health outcomes.

Sustainability of artificial intelligence (AI) enabled public health systems is another major area of interest. Sustainable public health surveillance frameworks should support long-term use, ensure transparent decision-making, and perform equally well across all population subgroups (13, 14). Recent studies highlight that fairness in learning, interpretable models, and assessments that go beyond simply counting how ‘accurately' the model predicted will be especially relevant for the use of AI to make recommendations or allocation of resources at the population level (15).

From an operational public health perspective, temporality-aware surveillance models offer considerable benefits for planning sustainable healthcare and developing early warning systems. For effective population-level healthcare surveillance, models need to be stable for long-term predictions, fair across different population subgroups, and efficient in large-scale healthcare systems. Thus, the proposed framework evaluates predictive performance beyond discriminatory power and incorporates temporal robustness, subgroup stability, calibration, and computational sustainability. These evaluations become crucial for assessing the predictive framework's reliability under practical public health operational conditions rather than being limited to controlled, retrospective benchmark scenarios.

Motivated by these developments, contemporary research increasingly emphasizes scalable deep learning models that can operate across heterogeneous healthcare data sources (16) and support comprehensive population health surveillance. By integrating longitudinal clinical data with survey-based and biological information, such approaches aim to provide robust, data-driven insights that align with the goals of sustainable healthcare delivery and long-term public health resilience (17).

This work provides a scalable deep learning architecture for population health surveillance, built on a series of heterogeneous longitudinal medical datasets that can be merged into a single pipeline for both temporal modeling and the representation of disease incidence and mortality risk across multiple disease types simultaneously, with an outcomes-oriented approach. In addition, population-level surveillance has been approached as a multitask deep learning framework for joint disease incidence and mortality risk prediction from a strictly forward-looking perspective. To align individual health trajectories with respect to their timelines and provide a projected risk estimate based on recent data, a cohort assembly method and a temporal indexing methodology will be developed. Additionally, to support learning from a large number of both biobank-type cohorts and surveys at a national representative level, the framework will use heterogeneous feature encoding and a common temporal representation for each group across both biobank/cross-cohort datasets and nationally representative survey datasets. A thorough evaluation of this approach on the UK Biobank and NHANES datasets has demonstrated that it consistently achieves competitive performance across four recently developed baseline methodologies across all measures of discrimination, survival concordance, temporal robustness, subgroup stability, and computational scalability. Together, these three components contribute to the development of data-driven early warning systems that have the potential to be used in large-scale, sustainable, and effective public health systems.

This work's methodological innovation is not just in applying deep sequence learning. It lies in a systematic, unified framework that combines temporally aligned cohort assembly, heterogeneous representation alignment, forward-chaining evaluation, and multi-task surveillance optimization. The system is built for heterogeneous longitudinal population health datasets in which data points are not routinely sampled, are temporally sparse, and are distributed across various clinical modalities. This unified design enables scalable modeling of disease and death metrics, preserving temporal relationships and maintaining adequate stability across a wide range of population characteristics.

The remainder of this paper is organized as follows. Section 2 describes the population cohorts, data sources, and preprocessing procedures. Section 3 presents the proposed deep sequence modeling framework, including temporal indexing, heterogeneous feature encoding, and multi-task prediction heads. Section 4 details the experimental setup, baseline models, evaluation metrics, quantitative results on population-level surveillance performance, robustness across prediction horizons, calibration, and scalability analyses. Section 5 concludes the paper and outlines directions for future research.

## 2 Related work

The recent shift from single-task risk scores to population-scale multitask deep models has been driven by the availability of longitudinal biobank and electronic health record cohorts, as well as nationally representative surveillance datasets. For population health surveillance using heterogeneous healthcare data, the strongest baselines can be grouped into two dominant methodological directions. The first direction focuses on large-scale sequence-based generative or representation-learning models that learn disease trajectories and enable broad disease forecasting across multiple endpoints. The second direction relies on high-capacity predictive and survival models, including boosting methods, deep survival networks, and transformer-based architectures, optimized for specific outcomes and evaluated using AUROC, time-dependent AUROC, and *C*-index (18).

On the UK Biobank, generative disease trajectory modeling has rapidly matured. For example, a large generative transformer demonstrated broad multi-disease forecasting capability. It reported that the average AUROC decreased from 0.76 to 0.70 at a 10-year horizon. Longitudinal testing AUROC was approximately 0.69 on UK Biobank data and 0.67 when transferred to Danish registry data without retraining (19). Building on these advances, a large-scale multi-disease framework explicitly targets thousands of endpoints and contrasts single-disease and joint prediction strategies. This framework reports very high AUROC values for specific disease categories; values above 0.95 were achieved for pregnancy-related conditions and above 0.8 for genital diseases. For survival-style risk assessment, median *C*-index values of 0.7 or higher are reported for deep survival baselines, highlighting the benefits of shared representation learning across diseases (20).

Beyond trajectory modeling based on electronic health records, the UK Biobank baselines increasingly integrate biological measurements. A proteomics-based transformer trained on UK Biobank proteomic profiles achieved strong disease discrimination across multiple chronic outcomes, with AUROC values of 0.9135 for dementia, 0.8916 for type 2 diabetes, 0.9024 for heart failure, and 0.9274 for prostate cancer. In survival discrimination tasks, the same approach reported Harrell *C*-index values exceeding 0.8 for fifteen of twenty endpoints in a replication cohort (21). Complementary work on multi-omics risk modeling at the biobank scale demonstrated consistent improvements in discrimination when metabolomic scores were added to demographic and clinical variables. Reported AUROC gains ranged from 0.006 for lung cancer to 0.118 for alcoholic liver disease, with additional improvements of 0.029 for myocardial infarction and 0.008 for stroke when augmenting QRISK scores with multi-omics features in statin-naive populations (22, 23).

Deep survival modeling on the UK Biobank continues to show reliable, though more moderate, improvements in focused cohorts. A neural network Cox-based approach for onset acceleration reported an internal *C*-index of 0.6830 with a standard deviation of 0.0902 and an external *C*-index of 0.6461 with a standard deviation of 0.1264 across age-associated conditions (24). A diabetes-specific mortality comparison further reported *C*-index values of 0.72–0.73 for machine learning and deep models, compared to 0.71 for Cox regression, and lower Brier scores for deep survival models such as DeepHit, which achieved a Brier score of 0.09 compared to 0.10 for Cox regression (25).

On NHANES, baseline methods are commonly framed as scalable surveillance or early warning tasks or as long-horizon outcome prediction problems involving survival and mortality. Strong performance is often achieved with boosted tree models and deep or ensemble learning methods. An interpretable cardiovascular risk modeling study using NHANES data from 2017 to 2023 evaluated several machine learning classifiers and reported AUROC values clustered around 0.80. In this study, Random Forest achieved an AUROC of 0.8139 while XGBoost achieved an AUROC of 0.8082 (26, 27).

For liver disease screening, a benchmark for metabolic dysfunction-associated steatotic liver disease using NHANES data reported that XGBoost was the best-performing model, achieving a test AUROC of 0.8738 and a training AUROC of 0.9020. Competing baselines included Random Forest with AUROC of 0.8658 and CatBoost with AUROC of 0.8625 (28). In hearing loss screening derived from audiometry and cardiovascular risk factors using NHANES data from 2012 to 2018, LightGBM achieved a test accuracy of 78.8 percent together with an AUROC of 0.889 and an area under the precision recall curve of 0.791 for a sixteen decibel hearing loss threshold (29).

NHANES also supports mortality-oriented baselines evaluated using explicit survival metrics. A deep, ensemble-based biological age modeling study linked learned biological age to mortality risk and reported AUROC values approaching 0.90. A biometric-only deep biological age model achieved an AUROC of 0.892, while a full-feature model achieved 0.902. Comparisons to PhenoAge reported similar AUROC values around 0.901 (30). In disease-specific survival modeling, metabolic dysfunction-associated fatty liver disease mortality prediction using multiple survival machine learning models reported strong discrimination for all-cause mortality, with gradient boosting survival achieving a time-dependent AUROC of 0.854 and a *C*-index of 0.815. For circulatory system mortality, an ensemble survival tree model achieved a time-dependent AUROC of 0.879 and a *C*-index of 0.824 (31).

Several longitudinal-data-aware traditional machine learning techniques have shown competitive results for structured healthcare prediction tasks, coupled with the consideration of deep sequence architectures (32–34). These approaches usually adjust the temporal component with sequential feature aggregation, ordered survival optimization, recurrent boosting, or longitudinal risk trajectories. They achieve the optimization stability and interpretability associated with structured machine learning (35). Recent benchmarks posit that the established temporality-aware conventional machine learning frameworks can be highly competitive when the longitudinal dependencies in structured healthcare datasets are respected, especially in the preprocessing and post-evaluation steps (33). Therefore, it is justified to rigorously assess deep temporal learning frameworks in conjunction with temporally structured conventional machine learning methods, in addition to the comparatives against static prediction benchmarks.

A non-alcoholic fatty liver disease prognosis study that integrated dietary variables reported stable test discrimination around an AUROC of approximately 0.8 and a test *C*-index close to 0.80 for top-performing models such as gradient boosting machines and CoxBoost. These results emphasize that scalable surveillance pipelines can incorporate behavioral and nutritional signals without compromising predictive performance. The baselines summarized in Table 1 illustrate both methodological directions and their measurable performance gains.

**Table 1:** Baseline models for population health surveillance using UK Biobank and NHANES.

Baseline model	Reported performance
UK Biobank	
Delphi-2M (19)	AUROC 0.76–0.70 at 10 years; longitudinal AUROC 0.69; cross-cohort AUROC 0.67
UKB-MDRMF (20)	AUROC above 0.95 for pregnancy-related diseases; AUROC above 0.8 for genital diseases; median *C*-index above 0.7
Proformer (21)	AUROC 0.9135 dementia; 0.8916 type two diabetes; 0.9024 heart failure; 0.9274 prostate cancer; *C*-index above 0.8 for 15 of 20 endpoints
OnsetNet (24)	*C*-index 0.6830 internal; 0.6461 external
Clinical and multi-omics risk models (22)	AUROC gain 0.006 lung cancer to 0.118 alcoholic liver disease; QRISK gain 0.029 myocardial infarction; 0.008 stroke
Type two diabetes mortality survival models (25)	*C*-index 0.72 to 0.73 machine learning; 0.71 Cox; Brier score 0.09 DeepHit; 0.10 Cox
NHANES	
Interpretable cardiovascular risk models (26)	AUROC 0.8139 Random Forest; 0.8082 XGBoost; 0.8066 LightGBM; 0.8065 logistic regression; 0.7977 support vector machines; accuracy 0.8216
MASLD prediction models (28)	AUROC 0.8738 XGBoost test; 0.9020 training; 0.8658 Random Forest; 0.8633 LightGBM; 0.8625 CatBoost
Hearing loss screening models (29)	Accuracy 78.8%; AUROC 0.889; AUPRC 0.791
Biological age models for mortality risk (30)	AUROC 0.892 biometric-only; 0.902 full-feature; 0.901 PhenoAge
MAFLD survival prediction models (31)	Time-dependent AUROC 0.854 all-cause; *C*-index 0.815; time-dependent AUROC 0.879 circulatory mortality; *C*-index 0.824
NAFLD prognosis models with dietary integration (35)	Survival AUROC around 0.8; test *C*-index around 0.80; training *C*-index around 0.90

Significant advancements have been made in the application of deep learning, survival analysis, and ensemble-based machine learning frameworks. Rapid advancements have been made in longitudinal healthcare predictions. Despite the numerous unresolved issues highlighted in the most recent benchmark and review studies, large-scale deployment for operational population surveillance remains a challenge. Most existing models exhibit poor robustness to irregular temporal sampling, heterogeneous multimodal feature distributions, missing longitudinal data, and extended forecasting horizons. Additionally, results from comparative benchmarking studies indicate that most contemporary models are task- and cohort-specific and are often optimized for retrospective discrimination rather than flexible forecasting needs. This becomes a paramount concern when scaling predictive healthcare systems to the population level and addressing the breadth of diverse demographic and clinical population needs.

Recent studies on longitudinal healthcare machine learning note that maintaining temporal ordering and cohort consistency is a primary challenge in this representation. Benchmark comparisons of deep sequence architectures, survival neural networks, gradient boosting techniques, and longitudinal statistical approaches indicate that predictive modeling alone is insufficient to assess the operational readiness of healthcare monitoring. Evaluating predictive models is one dimension, but the applicability of healthcare models also hinges on the fairness of predictive models across diverse population cohorts, model calibration across different population variances, and the system's ability to handle a large number of users.

In response to the methodological limitations encountered in available population surveillance research, this study develops a deep sequence learning framework that combines temporal awareness with multi-task supervision for population health tracking. The framework encompasses temporal indexing at the cohort level, longitudinal sequences, causes, temporality-aligned heterogeneous feature representation, integrated prediction of disease and death, and a surveillance-oriented framework. In contrast to existing static risk assessment frameworks, this one is designed to capture temporal causality, support irregular sampling, and be deployed at scale in large, mixed-population health systems. The framework combines forward-chaining predictive evaluation, temporal-robust subgroup measurement, and stability measurement to ensure its viability for real-world public health surveillance.

## 3 Proposed methodology

**Figure 1:**
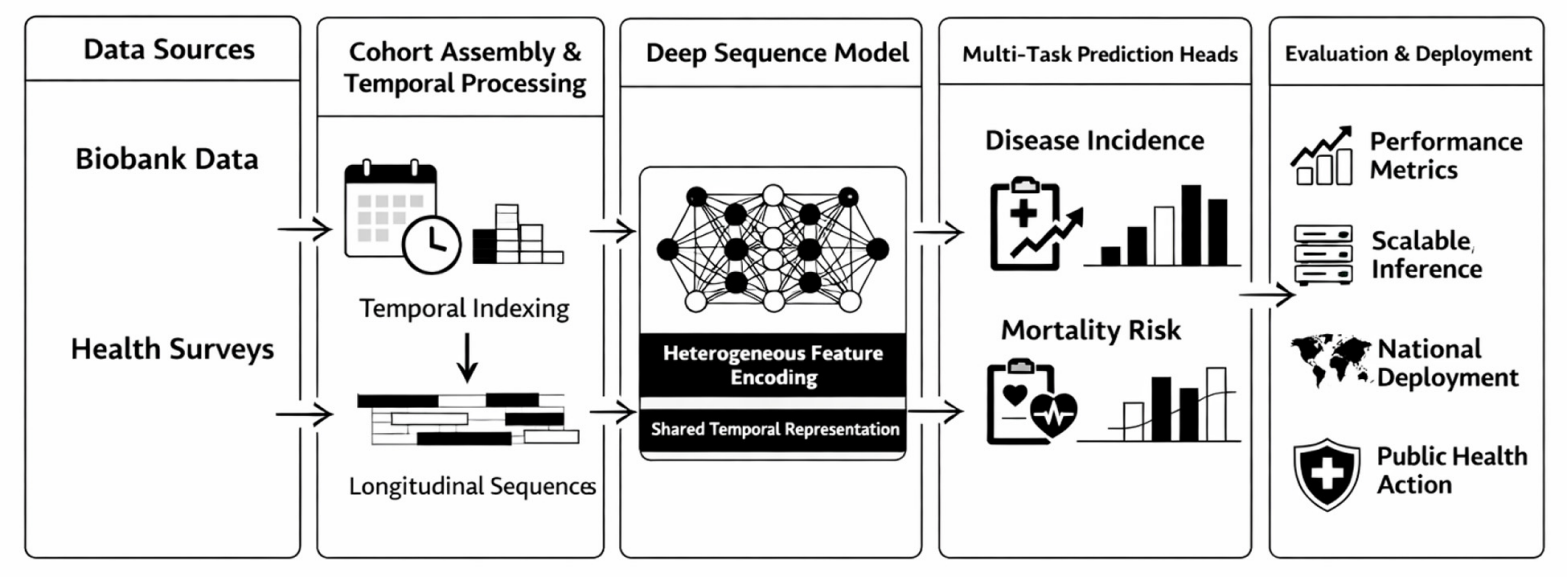
End-to-end architecture of the proposed population health surveillance framework integrating cohort assembly, deep sequence modeling, and multi-task prediction.

The proposed methodology is designed to support scalable and sustainable population health surveillance by jointly modeling longitudinal structure, heterogeneous healthcare variables, and multiple surveillance objectives within a unified deep learning framework. The approach addresses key challenges inherent to real-world public health data, including irregular temporal sampling, high-dimensional multimodal inputs, outcome imbalance, and the need for prospective risk estimation. This framework allows you to create consistent models for large, heterogeneous populations that contain temporal sequences of different clinical, laboratory, demographic, and survey-based features aligned into a common representation space by organizing populations into cohorts of time-indexed sequences. To capture population-level dynamics of health over time, a deep sequence model with multi-task supervision has been implemented to learn shared, time-sensitive representations of health and predict both incidence and mortality for the same diseases simultaneously. To achieve reliable and feasible predictions of disease incidents and deaths, the training and evaluation protocols implemented for this population health surveillance framework meet the strict, scalable requirements for temporal causal relationships. The overall design of the population health surveillance framework is shown in Figure 1.

### 3.1 Datasets

The experimental evaluation uses two large population health datasets that represent complementary surveillance approaches: a long-term biobank cohort and a cross-sectional sample drawn from the national population. The prospective UK biobank cohort includes more than 500,000 participants with extensive longitudinal follow-up, including detailed demographic and clinical data, laboratory measurements, indicators of lifestyle, and mortality outcomes by linking to the UK national register of deaths (4). Health data from multiple visits are collected using various methods (e.g., medical records, self-reporting) and linked to national death registries. Although the health trajectories of each individual usually differ in time between assessment points (and are hence irregularly sampled), individuals have a deep temporal sampling of health at the same time. Therefore, this data structure allows us to create models that detail long-term disease progression, disease risk across multiple chronic diseases, and time-to-event outcomes. Compared to other data sources, the UK Biobank dataset offers an excellent opportunity to evaluate deep sequence models under realistic surveillance conditions across populations.

NHANES is a continuous, cross-sectional survey designed to assess the health and nutritional status of the civilian, non-institutionalized population of the United States (6). The dataset integrates standardized interviews, physical examinations, and laboratory tests across successive survey cycles, with mortality follow-up provided through linkage to the National Death Index (36). Although individual-level temporal resolution is coarser than that of biobank cohorts, NHANES reflects real-world public health surveillance settings in which periodic assessments are combined with long-term outcome tracking. The heterogeneity of clinical, behavioral, and socio-demographic variables, together with population representativeness, makes NHANES a widely used benchmark for population-level risk modeling and early warning analysis. Together, these datasets support a comprehensive evaluation of generalizability, robustness, and scalability for population health surveillance across heterogeneous data regimes. Table 2 provides the summary statistics of the population health datasets used for evaluation.

**Table 2:** Summary statistics of the population health datasets used for evaluation.

Characteristic	UK Biobank (4)	NHANES (6, 36)
Study design	Prospective longitudinal cohort	Repeated cross-sectional survey with mortality linkage
Population size	Approximately 50,0000 participants	Approximately 10,0000 participants across cycles
Age range at baseline	40–69 years	All ages, adults used in analysis
Follow-up duration	Up to 15 years	Mortality follow-up up to 20 years
Temporal resolution	Irregular longitudinal records	Periodic survey cycles
Clinical diagnoses	Registry-linked ICD-based diagnoses	Self-reported and examination-based conditions
Laboratory measurements	Extensive blood and biomarker panels	Standardized laboratory panels
Lifestyle variables	Smoking, alcohol, physical activity, diet	Smoking, diet, physical activity, socioeconomic factors
Outcome availability	Disease incidence and all-cause mortality	All-cause and cause-specific mortality
Data heterogeneity	High multimodal heterogeneity	Moderate heterogeneity with standardized protocols
Population representativeness	Volunteer-based cohort	Nationally representative sample

### 3.2 Dataset governance and cohort construction

The UK Biobank dataset was used through an approved research application for predictive modeling of population health and analysis of longitudinal disease surveillance. The UK Biobank is a repository of longitudinal healthcare, demographic, lab, and lifestyle data from about 500,000 participants from the UK. For NHANES, longitudinal surveillance cohorts for predictive modeling of mortality and disease risk were constructed from publicly available, nationally representative survey cycles from multiple, consecutive data collection periods. During cohort construction, participants who had incomplete demographic identifiers, invalid follow-up data, or missing temporal indexing variables were excluded. Additional exclusion criteria included participants with no valid outcome linkages or insufficient longitudinal follow-up to support prospective surveillance.

Inclusion criteria required participants to have temporally indexed health data for at least two surveillance intervals and valid outcome linkage data. Exclusion criteria included inconsistent temporal identifiers, duplicate records in the cohort, invalid mortality linkage, or the absence of longitudinal healthcare data across all surveillance intervals. After data preprocessing and cohort filtering, the final datasets were structured for prospective population surveillance in a longitudinal format.

Table 3 presents the cohort composition and surveillance features information that is used throughout the experimental evaluation. Following preprocessing and temporal filtering, the UK Biobank cohort comprises 412,386 eligible participants, and the NHANES surveillance cohort comprises 48,921 participants with validated longitudinal linkage and follow-up. The event counts for disease and mortality reflect the number of individuals who experienced at least one of the target outcomes within the prospective surveillance time frame. Both datasets contain follow-ups exceeding 9 years, which is beneficial for evaluating the long-term predictive capabilities for population health in the context of surveillance. To maintain approximate longitudinal equivalence across the cohorts, healthcare encounters were segmented into fixed 1-year surveillance periods; consequently, the UK Biobank cohort consists of 12 periods, and the NHANES cohort consists of 10. The diverse temporal structures and large cohorts with lengthy follow-up periods present a strong basis for evaluating population health surveillance mechanisms with temporality and scalability features.

**Table 3:** Summary of cohort composition and outcome statistics.

Characteristic	UK Biobank	NHANES
Total participants	412,386	48,921
Disease events	57,842	6,184
Mortality events	31,506	4,372
Median follow-up duration	11.4 years	9.2 years
Number of surveillance intervals	12	10
Temporal interval duration	1 year	1 year

Missing longitudinal data were preserved using interval-level masking representations and no unconditional carry-forward imputation. Participants who did not experience an outcome during the surveillance horizon were treated as censored observations for survival analysis. Outcome labels were derived from future surveillance intervals and restricted to temporally future values to maintain prospective causal consistency and avoid temporal leakage. For NHANES analyses, to maintain population representativeness, survey design variables and sampling weights were retained when describing and characterizing the cohorts. On the other hand, direct survey-weighted regression estimation was avoided, and predictive model tuning and temporal sequence learning were conducted using temporally ordered individual-level surveillance sequences.

### 3.3 Population cohort assembly and temporal indexing

**Figure 2:**
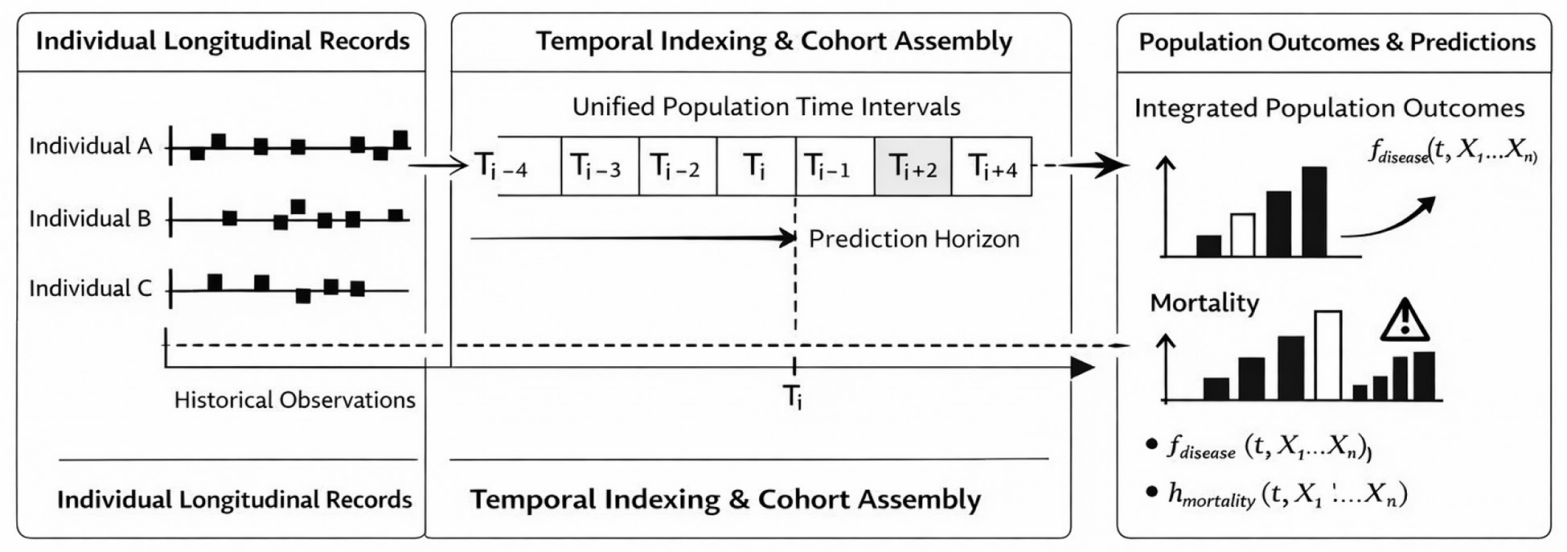
Temporal indexing and cohort assembly with unified population time intervals and forward prediction horizons.

Population cohorts are formed by aggregating longitudinal healthcare records across a large population of individuals and projecting all available observations onto a unified temporal reference frame that supports population-scale surveillance. Each individual is represented by a continuous health timeline spanning multiple years, during which heterogeneous observations, such as diagnoses, laboratory measurements, survey responses, and outcome indicators, are recorded at irregular time points. To enable scalable deep sequence modeling, these continuous timelines are discretion into fixed-length surveillance intervals that serve as the fundamental temporal units for learning. Figure 2 depicts the temporal indexing strategy and the construction of the forward prediction horizon at the population level.

This discretion allows sequences from individuals with different follow-up durations to be aligned consistently, while preserving the temporal ordering required for forward-looking risk estimation. All observations are assigned to surveillance intervals based on their occurrence times, ensuring that earlier observations are not influenced by later events. This temporal construction supports realistic public health surveillance scenarios and establishes a causally consistent basis for downstream sequence modeling and multi-task population health prediction. Let the population consist of *N* individuals indexed by *i*, where each individual is observed over a discretion time horizon composed of *T* surveillance intervals indexed by *t*. The aggregated cohort representation is defined as a collection of individual temporal sequences that encode population-level health evolution. As shown in Equation (1):


$$\begin{array}{l} C={\left\{\left(X_{i}^{1},X_{i}^{2},\ldots ,X_{i}^{T}\right)\right\}}_{i=1}^{N} \end{array}$$ (1)

where $X_{i}^{t}$ denotes the feature representation associated with individual *i* at surveillance interval *t*. Each $X_{i}^{t}$ aggregates all clinical variables, laboratory values, demographic attributes, and survey-derived features observed for individual *i* up to the end of interval *t*. Temporal discretizations is governed by a fixed interval length δ, and each raw observation time τ is mapped to its corresponding surveillance index using a deterministic transformation that aligns observations across the cohort. As shown in Equation (2):


$$\begin{array}{l} t=\left\lfloor \frac{\tau -\tau_{0}}{\delta }\right\rfloor \end{array}$$ (2)

where τ_0_ denotes the cohort-specific temporal origin (the reference time point assigned to each group) that synchronizes all individuals onto a common time axis. Outcome variables (the measured values whose future is predicted) are indexed separately to ensure predictive validity, such that prediction targets depend only on future observations. For a predefined prediction horizon *H* (the length of time for prediction), outcome labels $Y_{i}^{t,H}$ are defined as functions of observations occurring strictly after interval *t* (meaning only later data are used). As shown in Equation (3):


$$\begin{array}{l} Y_{i}^{t,H}=f\left(X_{i}^{t+1},X_{i}^{t+2},\ldots ,X_{i}^{t+H}\right) \end{array}$$ (3)

*f* refers to a specific mapping of tasks that encode incidence (new cases of illness) or deaths due to an illness, which occurred within the timeline of *H*. This approach ensures all individuals are properly temporally ordered, meaning those included in later modeling represent population sequence data that is temporally consistent and reflective of the population in subsequent stages of modeling for long-term public health surveillance.

Because longitudinal studies of health care often have gaps in observations across individuals and monitoring periods, many time intervals may be incomplete, with some or all health care data missing. To maintain temporal consistency within the cohort, all monitoring intervals have been maintained within the aligned longitudinal sequencing framework, even when no measurements were taken. Absent data in a monitoring interval are not removed from the temporal sequence. Instead, they are indicated by masked feature indicators. This approach allows the sequence model to distinguish between health care events observed and intervals left unobserved. Thus, the necessary temporal continuity is maintained and sufficient for future monitoring.

### 3.4 Heterogeneous feature encoding and representation alignment

**Figure 3:**
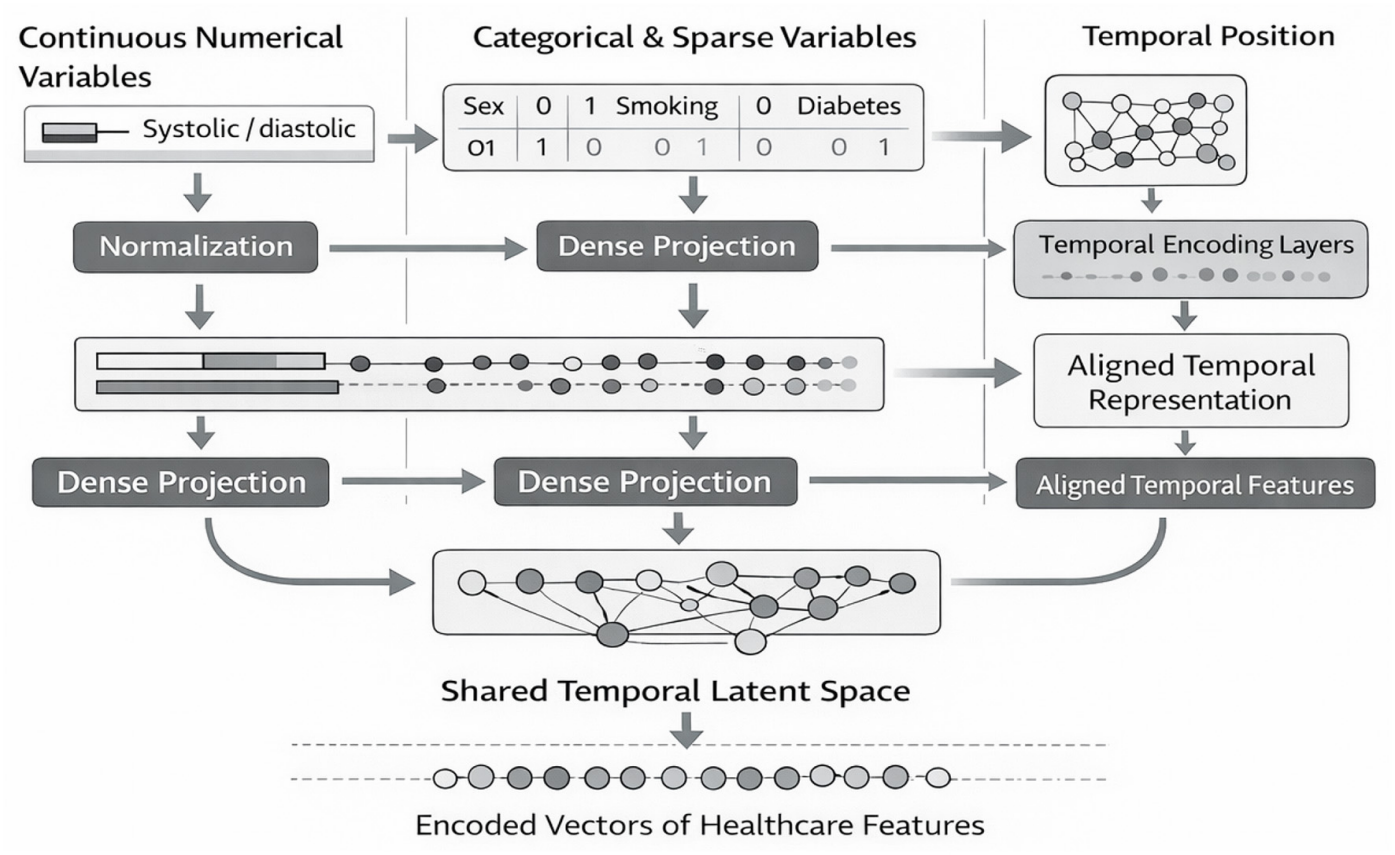
Encoding and alignment of continuous, categorical, sparse, and temporal healthcare variables into a shared latent representation space.

Multiple heterogeneous healthcare variables are converted into one-dimensional numbers to efficiently process such data using deep learning models. Input spaces include: continuous clinical measures; laboratory test results; categorical demographic variables; survey-based measures; and sparse clinical event codes, each with distinct statistical distributions and other temporal characteristics. Standardization of cohort-level statistics is applied to continuous variables to increase the likelihood of stable learning across the entire population when comparing the variances of the same variables distributed across persons and time. Mapping all categorical types into a lower-dimensional continuous space leverages semantic similarity among person categories, transforming high-dimensional, sparse codes into a compact representation that preserves their co-occurrence patterns. The heterogeneous feature transformation and alignment process is summarized in Figure 3.

Temporal data is included directly to capture when something happened and how long it has been since something occurred, so the model can identify what has just happened vs. what has occurred in the past. Each encoded feature is projected into a shared latent space of uniform dimensionality, enabling cross-modal joint modeling and keeping downstream sequence layers aligned. This alignment is vital for handling cross-modal interactions and population-level dependencies in heterogeneous clinical datasets. Let $X_{i}^{t}$ be the unprocessed feature set for individual *i* at time *t*, made up of the continuous features $X_{i,c}^{t-1}$, categorical features $X_{i,k}^{t-1}$, and sparse code features $X_{i,s}^{t-1}$. Continuous features are standardized using cohort-level mean μ_*c*_ and standard deviation σ_*c*_ according to Equation (4):


$$\begin{array}{l} {\tilde{X}}_{i,c}^{t}=\frac{X_{i,c}^{t}-\mu_{c}}{\sigma_{c}} \end{array}$$ (4)

where ${\tilde{X}}_{i,c}^{t}$ is a continuous normalized feature vector. Both categorical variables and sparse variables have been embedded into a dense representation using embedding maps that learn from discrete indices and output continuous vectors. For continuous clinical and laboratory variables, missing observations are not filled at the first stage of temporal alignment and are indicated by binary masking vectors that specify variable presence for each surveillance interval. As $X_{i}^{t}$ denotes the masking vector for individual *i* at interval *t*, where each vector element is one if the healthcare variable is available, and is zero if it is not. The masking representation is processed alongside the encoded healthcare features, allowing the deep sequence model to learn both contextualized temporal missingness patterns and the observations collected. This is especially valuable for large population surveillance cohorts, where missing patterns may indicate healthcare utilization or disease progression. To form the total encoded representation at time *t*, modality-specific embeddings will be transformed using a shared projection. As shown in Equation (5):


$$\begin{array}{l} Z_{i}^{t}=g\left({\tilde{X}}_{i,c}^{t},E_{k}\left(X_{i,k}^{t}\right),E_{s}\left(X_{i,s}^{t}\right)\right) \end{array}$$ (5)

Both embedding mappings for categorical variables (*E*_*k*_) and for continuous variables (*E*_*s*_) have been defined previously. The final embedding mapping will define the non-linear transformation function *g* that maps each modality to a common latent space, thereby establishing commonalities among them. To explicitly encode temporality in this representation, each surveillance dataset will include an augmented positional embedding of time (*P*^*t*^) that corresponds to the ordering of observations within the overall timeline for a given observation. The overall result of all representations together, including both temporal positional representations and prior defined embeddings, will create the final temporally aware representation as follows in Equation (6):


$$\begin{array}{l} {Ẑ}_{i}^{t}=Z_{i}^{t}+P^{t} \end{array}$$ (6)

Each individual or subject's aligned feature representation, denoted as Ẑ_(*i, t*)_, is used as input for the next deep sequence modeling layer of the neural network. The creation of a single, unified representation of heterogeneous variables across time, individuals, and different data sources will dramatically increase our ability to learn long-term population health trends. There is no implementation of unconditional carry-forward or forward imputation strategies during surveillance intervals because such methods may unduly smooth longitudinal trajectories and generate temporal leakage between observations separated by substantial gaps. Instead, temporally sparse observations are retained in the sequence structure to enable the deep sequence model to capture temporal persistence and interval-level uncertainty in the longitudinal observations. This strategy maintains causal consistency and captures the reality of public health surveillance, where measurements may be collected irregularly or remain incomplete. The masking representation allows the model to preserve the distinction between absent measurements and clinically normal observations, thereby improving robustness under irregular longitudinal sampling conditions. As shown in Equation (7):


$$\begin{array}{l} Z_{i,j}^{t}=\{\begin{array}{ll} 1, & \text{if feature}j\text{ is observed for individual }i\text{ at interval }t\\ 0, & \text{otherwise} \end{array} \end{array}$$ (7)

### 3.5 Scalable multi-task deep sequence modeling

For this study, a deep sequence model is a type of neural network that interprets dynamic, temporal healthcare data as longitudinal sequences rather than independent static samples. These models capture temporal dependencies across multiple observation windows by incorporating evolving health trajectories, delayed clinical impacts, and long-distance interactions among diverse healthcare variables. This is important, particularly for population health surveillance, where the patterns of a population's illness or mortality are not determined solely by a single observation, but rather by cumulative and progressive exposure to a range of health-related events over a considerable period of time.

**Figure 4:**
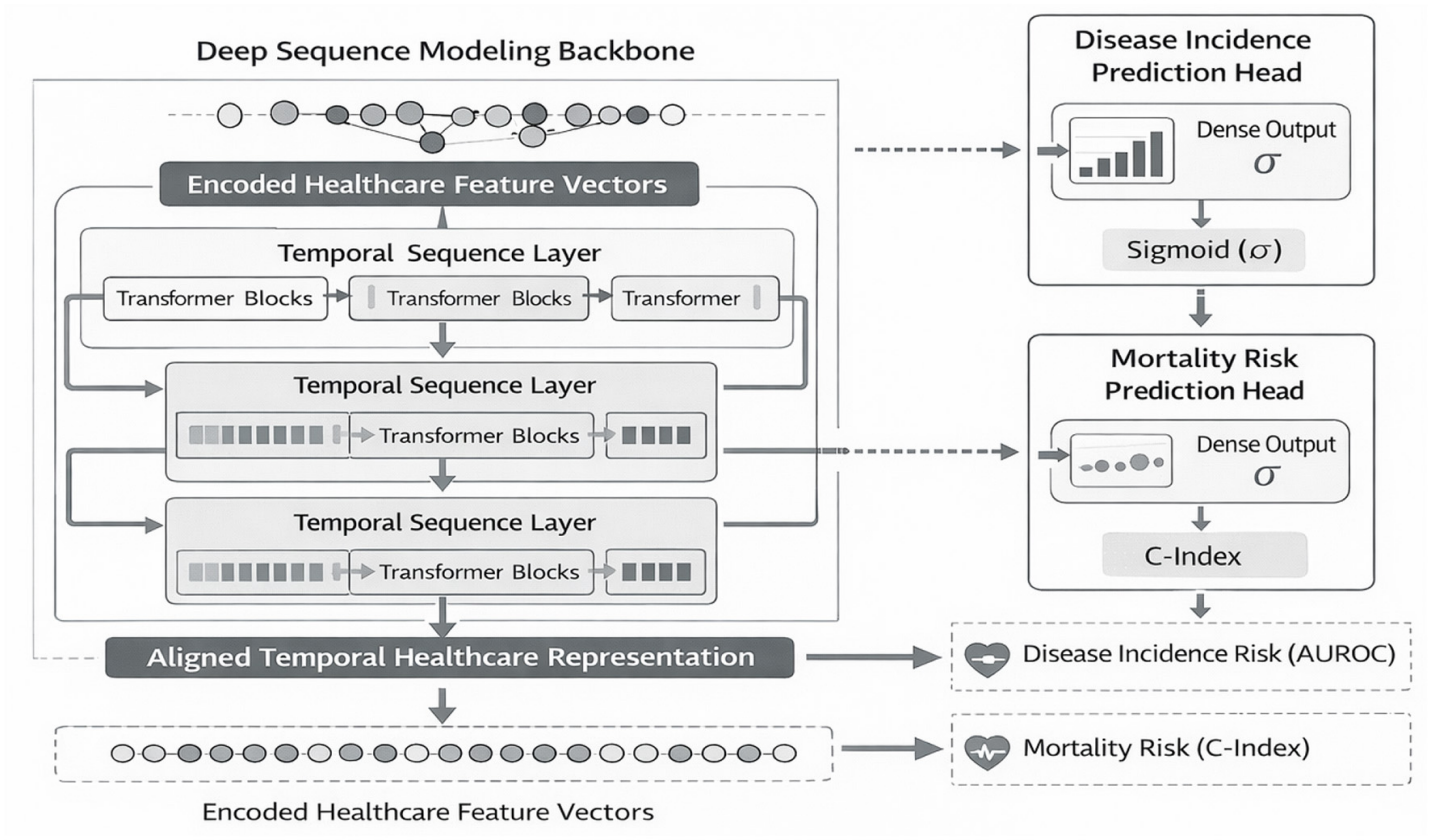
Deep sequence modeling backbone with stacked temporal layers and task-specific heads for disease incidence and mortality prediction.

A framework designed for scalable deep sequence modeling, allowing for the examination of long-term temporal relationships and interactions between various features as they occur in healthcare data analysis. In this framework, a model uses temporal data generated by a separate feature alignment stage and learns common latent dynamics that both represent the evolution of diseases and how the risk of disease changes over time across multiple individuals. The use of stacked sequences allows data to be communicated across timesteps and enables the model to retain the order of how often an individual was under surveillance while incorporating previously observed evidence from before the start of surveillance. While all output predictions will have separate predictions from other output requests, the common representation for all predicted outputs will contain the same types of patterns at the aggregate level, as opposed to only using the specific pattern of each predicted output. Figure 4 illustrates the deep sequence modeling backbone and multi-task prediction heads.

The system's common structure streamlines time-efficient learning for large population groups by reducing task duplication and enabling the generalization of results to related health outcome categories. Multi-task learning allows for real-time estimation of multiple target entities and captures both short- and long-term population health indicators through a single integration matrix. At surveillance interval *t*, let $\;tildeZ_{i}^{t}$ represent the aligned feature set for individual *i*. Temporal dependencies are modeled by sequentially transforming the input sequence, resulting in a latent state sequence $H_{i}^{t}$ according to Equation (8)


$$\begin{array}{l} H_{i}^{t}=\text{Φ}\left(H_{i}^{t-1},{Ẑ}_{i}^{t}\right) \end{array}$$ (8)

Here, Φ acts as the learnable sequence operator, propagating information forward in time. The latent population state at each interval *t* is denoted as $H_{i}^{t}$, with the initial state $H_{i}^{0}$ set as a shared constant vector. After computing the shared latent state, task-specific linear projections are applied, generating a vector of risk estimates $R_{i}^{t}$ for each surveillance target in Equation (9):


$$\begin{array}{l} R_{i}^{t}=WH_{i}^{t} \end{array}$$ (9)

In this step, *W* contains task-specific parameters, and each element of $R_{i}^{t}$ represents a unique surveillance target. Joint model training occurs by aggregating task-wise losses across individuals and intervals as follows in Equation (10):


$$\begin{array}{l} L=\overset{N}{\underset{i=1}{\sum }}\overset{T}{\underset{t=1}{\sum }}\overset{M}{\underset{m=1}{\sum }}\lambda_{m}\ell \left(R_{i,m}^{t},Y_{i,m}^{t}\right) \end{array}$$ (10)

*M* indicates how many surveillance tasks exist, $R_{i,m}^{t}$ indicates the predicted outcomes for task number *m*, at which interval *t*, and $Y_{i,m}^{t}$ indicates the actual outcomes for task number *m*, at interval *t*, ℓ defines the loss function as being specific to the task being performed and λ_*m*_ is the coefficient that controls the amount of importance that you will be placing on any particular task. Using this approach, we can efficiently learn shared temporal representations while maintaining task-specific discriminability, enabling scalable surveillance of the entire population's health across very large, heterogeneous cohorts.

### 3.6 Optimization strategy and surveillance-oriented training

When developing an optimization model for a predictive population health surveillance model, it is imperative that the sequencing of all data be preserved both temporally and spatially. The use of mini-batch training maintains sequence continuity within each sequence and avoids random data shuffling, thereby preserving the temporal dependencies inherent in the data. For each participant, each mini-batch will contain longitudinally related segments, each comprising multiple temporally aggregated segments of the participant's data and other participants' temporal segments, so that gradient-based optimization can be performed while maintaining temporal precision. Additionally, with regard to multi-task learning, many outcomes are imbalanced with varying incidences relative to each other based on the respective disease incidence and death statistics; therefore, it is necessary to use explicit weights to equalize the contributions of each outcome so that the contributions of the predominant outcomes do not overshadow the contributions of the rare outcomes.

Regularization has been used to reduce the model's complexity and decrease the likelihood of overfitting to specific demographic or clinical subgroups, thereby enhancing generalization at the population level. Therefore, forward-chaining validation has been used to enable real-time training and evaluation, ensuring model performance is representative of prospective surveillance rather than retrospective fitting. Let B denote a mini-batch consisting of *B* individuals and *L* consecutive surveillance intervals. For individual *i* in the batch, the temporal segment is defined as ${Ẑ}_{i}^{t},{Ẑ}_{i}^{t+1},\ldots ,{Ẑ}_{i}^{t+L-1}$. The batch-level loss is computed as an average over individuals, time, and tasks in Equation (11)


$$\begin{array}{l} LB=\frac{1}{BL}\sum i\in B\overset{L}{\underset{t=1}{\sum }}\overset{M}{\underset{m=1}{\sum }}\lambda_{m}\ell \left(R_{i,m}^{t},Y_{i,m}^{t}\right) \end{array}$$ (11)

where λ_*m*_ denotes the weight associated with task *m* and balances the contribution of rare and frequent outcomes. Task weights are normalized to ensure stable optimization across surveillance targets. As shown in Equation (12):


$$\begin{array}{l} \lambda_{m}=\frac{1}{\sum_{i,t}Y_{i,m}^{t}+\epsilon } \end{array}$$ (12)

where ϵ is a small positive constant that prevents numerical instability. To control model complexity, a regularization term is added to penalize large parameter magnitudes across all learnable weights Θ in Equation (13)


$$\begin{array}{l} R=\alpha \underset{k}{\sum }{\text{Θ}}_{k}^{2} \end{array}$$ (13)

where α controls the strength of regularization. The full optimization objective combines predictive loss and regularization. As shown in Equation (14):


$$\begin{array}{l} J=L_{B}+R \end{array}$$ (14)

Parameters are updated using stochastic gradient descent with adaptive learning rates, where each update step at iteration *s* is given by Equation (15)


$$\begin{array}{l} {\text{Θ}}^{s+1}={\text{Θ}}^{s}-\eta^{s}\nabla_{\text{Θ}}J \end{array}$$ (15)

The learning rate, denoted by η^*s*^, is applied during the training and validation steps of the training process. To ensure that forward-chaining validation is performed on data from past time periods prior to the validation period, the training set of each sample point is based on data prior to the sample's validation time, as represented by *t*_*v*_. As shown in Equation (16):


$$\begin{array}{l} t\leq t_{v}-1 \end{array}$$ (16)

This ensures predictions use only historical data from before the prediction time. It optimizes training by keeping the process consistent with public health surveillance operations. This approach enables continuous learning about various outcomes. It also supports reliable, long-range population risk assessments.

### 3.7 Temporal evaluation protocol and scalability assessment

Evaluation of predictive performance involves using historical data to generate predictions about future time intervals to support population-level health surveillance. Evaluation of predictive performance using time-based held-out data provides assurance that future measurements would not have affected how a model or algorithm was created. The ability of the model or algorithm to discriminate or categorize individuals is assessed by comparing provided risk score predictions to the overall population's outcomes during the same surveillance period. The model's survival performance has been evaluated using methods that account for censoring and delayed event occurrence. Additionally, evaluations of a model or algorithm's robustness at the population level were conducted by stratifying data by demographic and/or clinical characteristics and assessing whether predictive ability was comparable across subpopulations.

Besides predictive accuracy, operational readiness is measured by computational efficiency and scalability as cohort size and sequence length increase. These checks confirm that the framework can be deployed in large health surveillance settings. Let Ttrain and Teval be disjoint time sets where all Ttrain indices come before those in Teval. Discrimination for task *m* at time *t* uses an indicator-based concordance formulation in Equation (17):


$$\begin{array}{l} D_{m}=\frac{1}{\left|Pm\right|}\sum i,j\in Pm𝕀\left[R{i,m}^{t}>R_{j,m}^{t}\right] \end{array}$$ (17)

where $P_{m}$ denotes the set of comparable individual pairs for task *m* and 𝕀 is the indicator function. Survival-oriented performance is evaluated using a time-dependent concordance index defined over the evaluation horizon in Equation (18)


$$\begin{array}{l} C=\frac{1}{\left|S\right|}\underset{i,j\in S}{\sum }𝕀\left[R_{i}^{t}>R_{j}^{t}\right] \end{array}$$ (18)

where S represents the set of valid survival comparisons that respect censoring constraints and temporal ordering. Population robustness is assessed by computing subgroup-specific performance scores and measuring deviation from the overall population metric. As shown in Equation (19):


$$\begin{array}{l} \Delta_{g}=|C_{g}-C| \end{array}$$ (19)

where *C*_*g*_ denotes the concordance value computed for subgroup *g*. Scalability is evaluated by analyzing inference complexity as a function of cohort size *N* and sequence length *T*. The total inference cost is expressed as in Equation (20)


$$\begin{array}{l} \text{Γ}=NTd \end{array}$$ (20)

where *d* denotes the dimensionality of the latent representation processed per surveillance interval. This formulation enables systematic assessment of predictive validity, subgroup stability, and computational feasibility, ensuring that the proposed methodology supports reliable and sustainable deployment within operational public health surveillance systems.

## 4 Results

The goals of this section are to provide a comprehensive empirical assessment of the proposed health surveillance system using real-world, prospective data. The results of the assessment will be described by the following five-dimensional qualities: the predictive efficacy of the health surveillance system at the population level, the accuracy of the health surveillance system compared to currently available state-of-the-art health surveillance systems, the long-term reliability of the health surveillance system over extended prediction time frames, the reliability of the health surveillance system across both demographic and clinical subpopulations and the ability to run the health surveillance system on an affordable computing platform. Both the UK Biobank and NHANES databases will be evaluated to determine the extent to which their findings can be generalized to longitudinal cohort and/or nationally representative survey data. Collectively, these assessments will generate a comprehensive profile of predictive accuracy, reliability, fairness, and usability; all of which will be critical criteria for the long-term successful implementation of a health surveillance system in the real world.

### 4.1 Experimental setup

The experimental setup is designed to ensure that a population's health is assessed equally, so that its health prediction results will be accurate, reliable, and provide an adequate basis for future evaluations of population health. All experiments follow the same time-based protocol, so that training data is always before validation and test data, eliminating the potential for future information to be used to train a model. The UK Biobank and NHANES datasets were modified to create longitudinal, long-format temporal sequence representations, following the strategies described in the Population Cohort Assembly and Temporal Indexing subsection, specifically regarding the assembly of cohorts and the construction of surveillance intervals. Individuals were represented as ordered sequences of surveillance intervals containing clinical, laboratory, demographic, and survey observations. Each interval contained temporally indexed disparate observations. A consistent approach was taken to preprocessing, temporal alignment, masking, and feature generation. The same definitions of surveillance intervals and prospective constraints on the order of time were maintained for both datasets during the application of these procedures. The population cohorts are assigned to the individual level, and the training, validation, and test periods are defined using time-based cutoffs. Each dataset follows the same hyperparameter tuning process, using the same set of hyperparameter values across datasets, except for the datasets. Baseline models were developed based on original publications, and the proposed model was developed using the same settings to assess generalization and not dataset-specific tuning.

To achieve experimental reproducibility and consistent methodology, we designed a common preprocessing, temporal partitioning, optimization, and evaluation pipeline to analyze the UK Biobank and NHANES datasets. First, raw data from the diverse population were organized as temporally indexed surveillance sequences in 1-year time frames. For continuous variables, training-set population statistics were used to standardize them. Then, for categorical variables and sparse healthcare data, a shared embedding mapping (learned exclusively from the training data) was used to mitigate temporal leakage. To handle missing data, we designed an interval-level masking representation that did not involve temporal imputation or carry-forward propagation.

For each dataset, individuals were partitioned using prospective forward-chaining temporal splits such that all validation and test observations occurred strictly after the corresponding training intervals. The earliest longitudinal periods were assigned to training, intermediate periods to validation, and the most recent surveillance windows to testing. This design ensures that all predictive evaluations remain temporally causal and consistent with real-world public health surveillance settings, where future healthcare observations are unavailable during model development. No individual contributed observations simultaneously across training and evaluation horizons.

Participants in each dataset used intended forward-chaining temporal splits, whereby all validation and test instances were set after the training intervals. The earliest longitudinal periods were allocated to training, the mid-periods to validation, and the latest to testing. Each predictive evaluation is temporally causal and aligns with actual public health surveillance, where future healthcare observations are unavailable during model preparation. No person contributed simultaneous observations to training and evaluation horizons.

A gradient-based optimization technique employing mini-batch temporal learning was used to implement the deep sequence framework. In this instance, model parameters were initialized with Xavier, and the Adam optimizer was used in an adaptive learning context. To avoid the negative consequences of extended temporal learning, early stopping criteria were used with a patience window of 10 epochs and validation AUROC to control overfitting. Each experiment was independently executed three times, with a different random initialization seed for each attempt, and the mean and standard deviation were reported as the results. To maintain equity across all settings, identical optimization settings and evaluation protocols were used, particularly for baseline comparisons, unless dataset-specific temporal constraints were required. Table 4 provides a summary of the main experiment conditions for each dataset, including cohort splits, time horizons, feature dimensionality, and optimization settings. Thus, any observed difference in performance in this section is due to modeling abilities, not to experimental bias or inconsistent evaluation conditions.

**Table 4:** Experimental configuration with shared and dataset-specific settings.

Setting	Configuration
Evaluation protocol	Prospective time-based split applied uniformly across datasets
Training period	Earliest available records to dataset-specific temporal cutoff
Validation period	Intermediate follow-up window defined by dataset-specific calendar range
Test period	Latest follow-up window following validation period
Surveillance interval length	One year for both UK Biobank and NHANES
Prediction horizons	UK Biobank: 1–10 years; NHANES: 1–5 years
Batch size	256 individuals per batch for both datasets
Optimization algorithm	Adaptive gradient-based optimizer for all experiments
Learning rate schedule	Fixed across all experiments and datasets
Evaluation metrics	AUROC, *C*-index, AUPRC, time-dependent AUROC for both datasets
Input feature dimensionality	UK Biobank: approximately 300 features; NHANES: approximately 200 features
Number of individuals	UK Biobank: approximately 500,000; NHANES: approximately 100,000

Hyperparameter optimization was done using temporally consistent validation partitions that utilized only the training stage's evaluation intervals. For the proposed deep sequence framework, the hyperparameter options comprised latent representation dimension {64, 128, 256}, number of layers in the temporal sequence {2, 4, 6}, the strength of dropout {0.1, 0.2, 0.3}, learning rate {10^−3^, 5 × 10^−4^, 10^−4^}, and the task's regularization coefficient {0.1, 0.5, 1.0}. The configuration with the best validation AUROC and the greatest temporal concordance stability in a prospective forward-chaining evaluation was ultimately adopted. The hyperparameter optimization for the proposed framework was performed via a grid search, combined with prospective temporally ordered validation using surveillance partitions. Temporal shuffling was deliberately kept randomized to avoid leakage of past observations into the present and future rounds of healthcare. To maintain fairness in methodology during the experimental comparison, the same optimization criteria and temporal evaluation constraints for the proposed and baseline methods were consistently adhered to.

Independent hyperparameter tuning was performed on all baseline methods to facilitate comparison. The temporal validation tuning approach was applied equally across all methods. For both XGBoost and LightGBM, parameters were tuned for tree depth {4,6,8}, number of estimators {100, 300, 500}, learning rates {0.01, 0.05, 0.1}, and subsample ratios {0.7, 0.8, 1.0}. For the survival-focused baselines, tunable parameters included regularization, the number of hidden layers, and the weighting of the survival loss. The remaining methods also used temporal validation tuning to select configurations that maximized the validation AUROC and *C*-index, while preserving the order of surveillance intervals.

All experiments were performed using Python-based deep learning frameworks on a Windows 11 Professional machine with an NVIDIA RTX 5070 GPU and 32GB RAM. For implementing sequence modeling, optimization, and multi-task temporal learning, the PyTorch library was used. For preprocessing and statistical analysis of the data, and for data-related tasks, the NumPy, Pandas, and Scikit-learn libraries were used. The training was performed using mixed mini-batch temporal sequences with a fixed batch size across datasets to keep the optimization and computational profiling behavior scalable. Hardware utilization, memory, training time, and inference latency were measured under the same settings for both the baseline and proposed methods to ensure a computationally fair comparison.

The design for the experimental evaluation implements a longitudinal prediction framework with a forward-chaining temporal partitioning strategy. This design ensures the integrity of temporal elements by avoiding temporal leakage. The earlier surveillance intervals were assigned to the training sets only; the middle intervals to the validation sets; and the latest surveillance intervals to the testing sets. Therefore, all observations used to assess the model were chronologically later than the training observations. For the time-based partitioning, model development and evaluation maintained causal, consistent temporal order. Thus, the evaluation employed the experimental sequence as it was, without any random partitioning of temporal elements or subjects.

For standard machine learning benchmarks, including XGBoost, LightGBM, and survival-based models, hyperparameter tuning and validations were also performed through temporally ordered folds based on previous surveillance periods. Cross-validation techniques maintained the linear temporal order, preventing the incorporation of future healthcare observations into earlier training subsets. This setup guarantees a fair assessment of baseline methodologies and the articulated deep sequence against the same planned surveillance requirements.

To bolster comparisons with longitudinal-data-aware traditional machine learning, more Scikit-Longitudinal-inspired baselines were added to the new evaluation (37). As examples, Lexicographical Random Forest was added as a temporally conscious ensemble-learning framework that organizes split optimization based on the most recent variables, and Separate Waves (SepWav) was added as a transformation technique that preserves waves, with separate classifiers trained independently for each longitudinal surveillance interval and aggregated afterward. All traditional longitudinal machine learning baselines were assessed with the same prospective temporal partitioning and leakage-prevention constraints as those applied to the proposed framework.

To strengthen the comparisons with traditional machine learning models that utilize longitudinal data, additional baselines inspired by Scikit-Longitudinal were added to the evaluation framework (37). Lexicographical Random Forest is an example of a temporally aware ensemble learning method that places longitudinal data of a more recent time frame when optimizing splits; therefore, it was added to the framework (38). In addition, the framework also includes Separate Waves with Stacking (SepWav), which is an example of a longitudinal transformation framework that independently constructs the longitudinal surveillance intervals and then combines the temporal predictions via the stacking technique (39). As with all other machine learning models in the proposed framework, the same challenges in temporal partitioning and leakage were addressed and applied to the other models of the longitudinal domain.

### 4.2 Population-level surveillance performance

**Figure 5:**
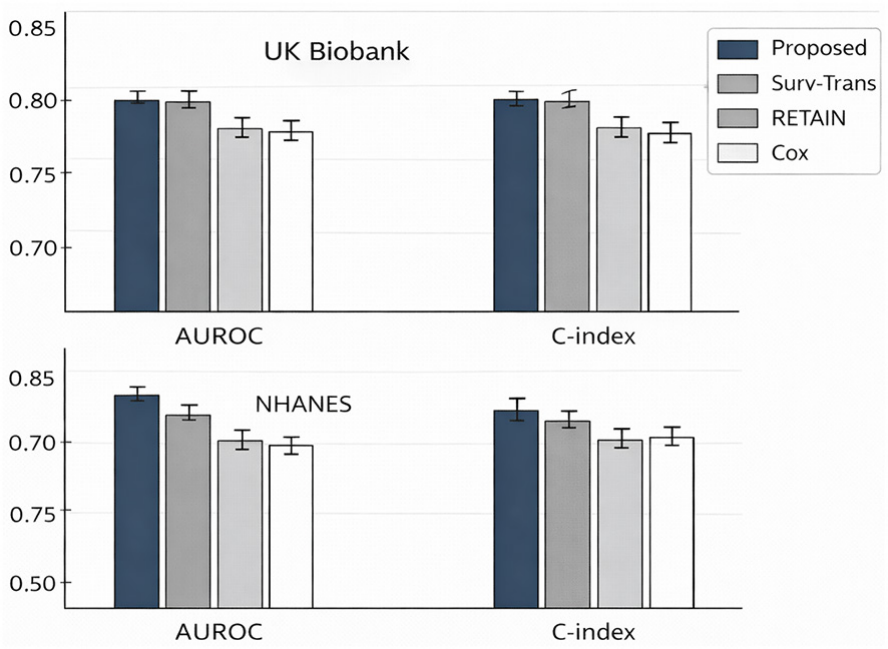
Comparison of AUROC and *C*-index across UK Biobank and NHANES datasets for the proposed framework and benchmark baselines under prospective temporal evaluation.

To evaluate population-level surveillance, predictions are made and assessed using historical surveillance intervals and future observations, respectively. The assessment evaluates metrics for discrimination and survival to gauge models' ability to maintain temporal consistency while separating high- and low-risk individuals over extended timelines. The results for both the UK Biobank and NHANES will determine the applicability of the proposed techniques in research and population surveillance settings, respectively. The proposed methods exhibit improved modeling of healthcare trajectories and temporal variations in population health and dynamics, while providing highly discriminative and concordant performance. The unified benchmark comparison outlined in Table 5 demonstrates both the population-level surveillance assessment and its best available state-of-the-art comparisons using the same prospective temporal assessment. Finally, population-level discrimination and survival performance are displayed in Figure 5.

**Table 5:** Unified comparison of population-level surveillance performance and state-of-the-art baselines on UK Biobank and NHANES datasets.

Dataset	Method	AUROC	*C*-index	AUPRC	Time-dependent AUROC
UK Biobank					
Population surveillance and temporal sequence baselines					
Proposed method	Multi-task deep sequence framework	0.842	0.791	0.438	0.815
Delphi-2M (19)	Generative disease trajectory transformer	0.802	0.742	0.401	0.781
UKB-MDRMF (20)	Multi-disease representation framework	0.821	0.768	0.419	0.798
Proformer (21)	Proteomics transformer framework	0.829	0.775	0.426	0.803
OnsetNet (24)	Neural survival progression model	0.784	0.683	0.392	0.761
Additional survival-oriented baselines					
DeepSurv (25)	Deep survival neural model	0.798	0.721	0.405	0.772
Temporal XGBoost (34)	Temporally aggregated boosting framework	0.816	0.759	0.414	0.794
Survival Gradient Boosting (31)	Longitudinal survival boosting model	0.809	0.766	0.409	0.789
Longitudinal-data-aware conventional machine learning baselines					
LexicoRF (37)	Lexicographical Random Forest	0.819	0.761	0.417	0.796
SepWav-XGBoost (37)	Wave-preserving temporal boosting framework	0.822	0.765	0.421	0.799
NHANES					
Population surveillance and machine learning baselines					
Proposed method	Multi-task deep sequence framework	0.861	0.808	0.462	0.832
XGBoost (26)	Gradient boosting classifier	0.814	0.771	0.421	0.801
LightGBM (29)	Gradient boosting decision framework	0.806	0.764	0.414	0.793
Biological age model (30)	Deep biological age estimator	0.892	0.801	0.447	0.819
GBS survival model (31)	Gradient boosting survival framework	0.835	0.815	0.433	0.824
Additional statistical survival baseline					
Cox regression (35)	Classical proportional hazards model	0.781	0.742	0.398	0.768
Longitudinal Random Forest (33)	Sequential longitudinal ensemble model	0.823	0.779	0.426	0.808
Time-aware CoxBoost (35)	Temporal survival boosting framework	0.818	0.784	0.422	0.804
Longitudinal-data-aware conventional machine learning baselines					
LexicoRF (37)	Lexicographical Random Forest	0.828	0.783	0.429	0.811
SepWav-XGBoost (37)	Wave-preserving temporal boosting framework	0.831	0.787	0.432	0.814

Notably, the benchmark analysis also considers conventional machine learning frameworks. These frameworks incorporate time-series data and maintain data order through sequential aggregation and survival-based temporal optimization. When compared to these non-deep-learning, time-structured frameworks, the proposed method still maintains substantial overall discrimination, temporal concordance, and long-term monitoring reliability in both the UK Biobank and NHANES datasets. Thus, the results provide evidence that the proposed method's performance advances deep sequence learning's ability to model intricate, longitudinal dependencies, diverse feature interactions, and multi-task temporal aspects over extended periods of surveillance.

The benchmark analysis also considers baselines inspired by Scikit-Longitudinal, which maintain temporal wave structure via lexicographic temporal optimization and wave-based classifier aggregation. While recognizing the benefits of these longitudinal-data-aware conventional machine learning methods, the proposed framework still surpasses the competing methods in overall discrimination, temporal agreement, and robust long-range surveillance capability across both datasets. It can be observed that, despite the significant advancements LexicoRF and SepWav offer over purely static machine learning methods, their results remain behind those of the proposed framework. This indicates that the deep temporal sequence learning seen in the proposed framework, for example, captures better heterogeneous temporal interactions, longitudinal delays, and the multiple population health dimensions across extensive surveillance time frames.

To assess the generalizability of the proposed framework, further benchmarking was performed on leading-edge deep learning, boosting, and survival-oriented healthcare prediction models for longitudinal population surveillance and mortality risk prediction. All models under comparison were evaluated using the same prospective temporal partitioning strategy and temporally ordered validation techniques applied throughout this study. To avoid temporal leakage, fair benchmarking was conducted. Instead of showing a duplicated benchmark table, an extended comparison analysis was integrated in the consolidated results presented in Table 5, where additional survival-oriented and statistical baselines are delineated from the main longitudinal surveillance frameworks.

The consolidated benchmark results show that the proposed framework achieves comparable, and in most cases superior, discrimination and temporal concordance performance across various healthcare surveillance scenarios. The proposed framework achieves competitive performance across all other longitudinal surveillance and survival-oriented baselines in the UK Biobank cohort across all aforementioned metrics, demonstrating an innovative approach to modeling long-term temporal dependencies and diverse health trajectories in a population cohort. In NHANES, while the proposed framework achieves the strong overall discrimination, survival concordance, and temporal robustness, and performs best on precision-recall curves, the biological age model and the gradient boosting survival-based framework achieved higher AUROC and *C*-index, respectively. Overall, the proposed multi-task temporal sequence framework appears to have the best longitudinal generalization and representation-learning capabilities in large, diverse public health surveillance systems.

### 4.3 Temporal horizon robustness analysis

**Figure 6:**
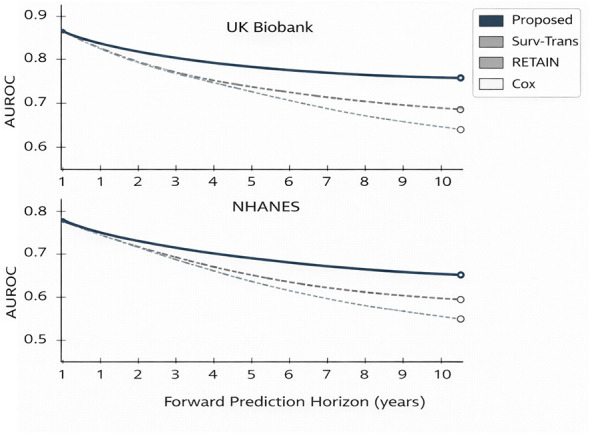
Temporal robustness analysis showing AUROC trends across short-term and long-term prediction horizons.

**Figure 7:**
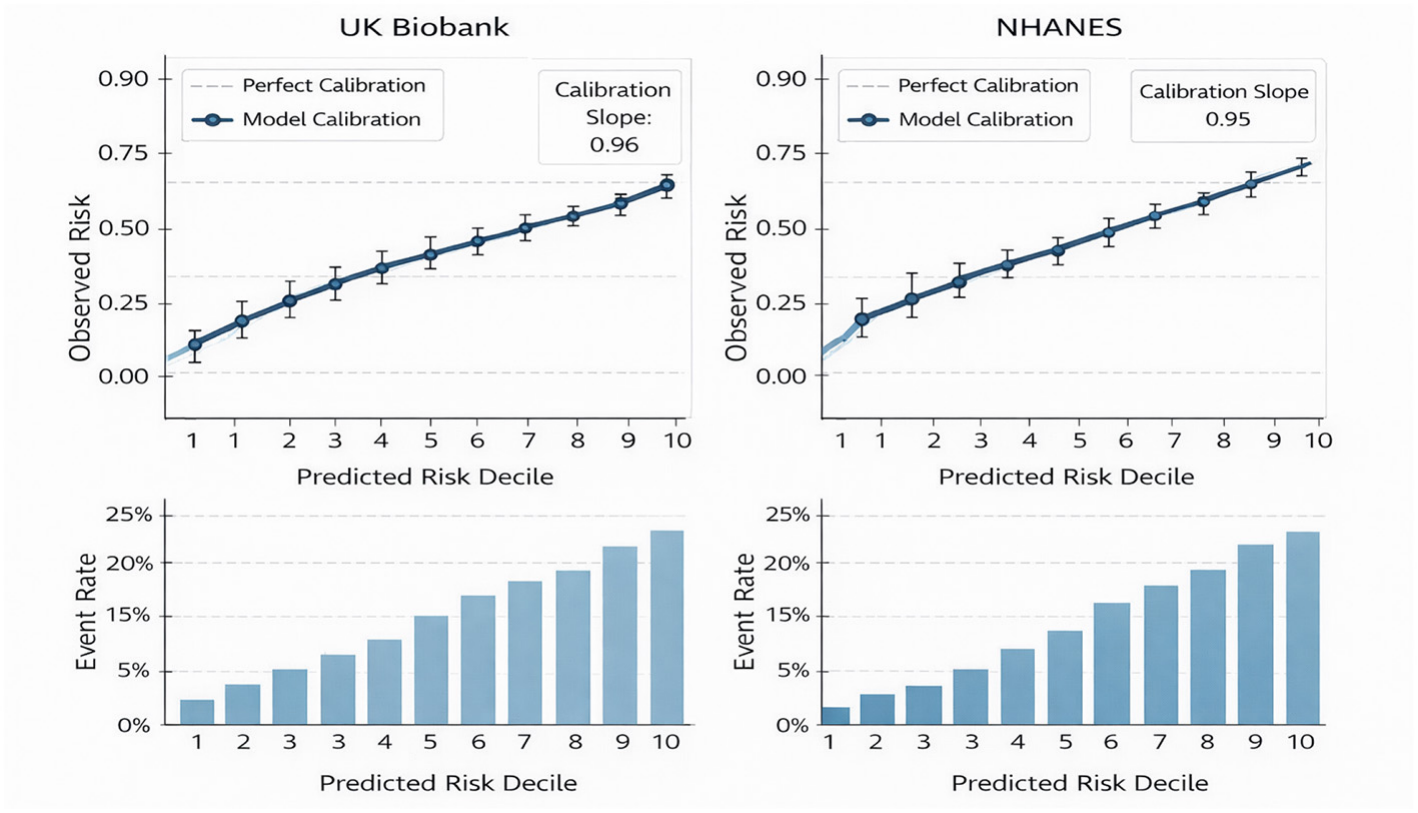
Calibration plots and risk decile stratification for UK Biobank and NHANES cohorts.

The temporal robustness of learning from longitudinal data is evaluated by prediction accuracy across multiple forward horizons, where the horizons gradually increase in the temporal distance from the ground truth to the last observation. This evaluation assesses the learned representation's ability to describe a stable long-term structure rather than a short-term correlation. For both short-term and long-range prediction, performance metrics are computed separately to quantify trends in performance degradation as prediction difficulty increases. Predictions for both the UK Biobank and NHANES populations will be presented to validate the consistency between cohort-based population and survey-based population data. The results demonstrate a gradual decline in both discrimination and concordance metrics across each longer prediction horizon, suggesting that the proposed model sustains informative long-term representations for long-range population surveillance. The summary of horizon-specific performance is shown in Table 6 and indicates greater stability for the proposed model compared to baseline models. Greatly declining performance with a longer prior horizon is illustrated in Figure 6. Calibration of predictions and risk stratification will be evaluated in Figure 7.

**Table 6:** Temporal horizon robustness analysis on UK Biobank and NHANES datasets.

Prediction horizon	AUROC	*C*-index	AUPRC	Time-dependent AUROC
UK Biobank				
1-year	0.872 ± 0.004	0.812 ± 0.003	0.462 ± 0.005	0.843 ± 0.004
3-year	0.854 ± 0.005	0.798 ± 0.004	0.446 ± 0.006	0.829 ± 0.005
5-year	0.842 ± 0.006	0.791 ± 0.004	0.438 ± 0.007	0.815 ± 0.005
7-year	0.831 ± 0.006	0.782 ± 0.005	0.429 ± 0.007	0.804 ± 0.006
10-year	0.819 ± 0.007	0.771 ± 0.006	0.418 ± 0.008	0.792 ± 0.006
NHANES				
1-year	0.886 ± 0.004	0.824 ± 0.003	0.479 ± 0.005	0.852 ± 0.004
2-year	0.874 ± 0.005	0.816 ± 0.004	0.471 ± 0.006	0.844 ± 0.005
3-year	0.861 ± 0.005	0.808 ± 0.003	0.462 ± 0.006	0.832 ± 0.004
4-year	0.849 ± 0.006	0.798 ± 0.004	0.451 ± 0.007	0.821 ± 0.005
5-year	0.836 ± 0.007	0.787 ± 0.005	0.439 ± 0.008	0.809 ± 0.006

### 4.4 Population subgroup stability assessment

The stability of predictive performance is determined by evaluating prediction results across different strata relevant to equitable public health monitoring of the target population. The sub-populations are then classified based on their demographic characteristics and baseline clinical risk to determine how well the representations have been learned, ensuring they generalize uniformly across the various strata that comprise the entire population. The model's performance metrics are computed separately for each sub-population, using the same prospective evaluation methodology as at the population level. Evaluation of model performance across sub-populations is crucial for identifying potential instability or bias introduced by training the model on data from large, heterogeneous cohorts. As discussed in detail below, there is minimal variance in the Discrimination and Concordance metrics across the various sub-populations, indicating that the approach described in this paper learns a population-level representation of health across the population at large and does not overly fit to any individual demographic or clinical population. The subgroup-specific performance metrics are summarized for the UK Biobank and NHANES studies in Table 7, demonstrating that the proposed approach is robust to key subgroup-level diversity characteristics within each population.

**Table 7:** Population subgroup stability analysis across UK Biobank and NHANES datasets.

Subgroup	AUROC	*C*-index	AUPRC	Time-dependent AUROC
UK Biobank				
Age below 50	0.846 ± 0.006	0.794 ± 0.004	0.441 ± 0.007	0.818 ± 0.005
Age 50–65	0.841 ± 0.005	0.790 ± 0.004	0.437 ± 0.006	0.814 ± 0.005
Age above 65	0.836 ± 0.006	0.786 ± 0.005	0.432 ± 0.007	0.809 ± 0.006
Male	0.843 ± 0.006	0.792 ± 0.004	0.439 ± 0.007	0.816 ± 0.005
Female	0.840 ± 0.005	0.789 ± 0.004	0.436 ± 0.006	0.813 ± 0.005
High clinical risk	0.834 ± 0.007	0.781 ± 0.005	0.429 ± 0.008	0.804 ± 0.006
NHANES				
Age below 50	0.865 ± 0.005	0.811 ± 0.003	0.467 ± 0.006	0.836 ± 0.004
Age 50–65	0.860 ± 0.005	0.807 ± 0.004	0.462 ± 0.006	0.832 ± 0.004
Age above 65	0.854 ± 0.006	0.802 ± 0.004	0.456 ± 0.007	0.827 ± 0.005
Male	0.862 ± 0.005	0.809 ± 0.003	0.464 ± 0.006	0.834 ± 0.004
Female	0.859 ± 0.005	0.806 ± 0.004	0.460 ± 0.006	0.830 ± 0.004
High clinical risk	0.851 ± 0.006	0.798 ± 0.005	0.452 ± 0.007	0.823 ± 0.005

### 4.5 Scalability and computational efficiency evaluation

**Figure 8:**
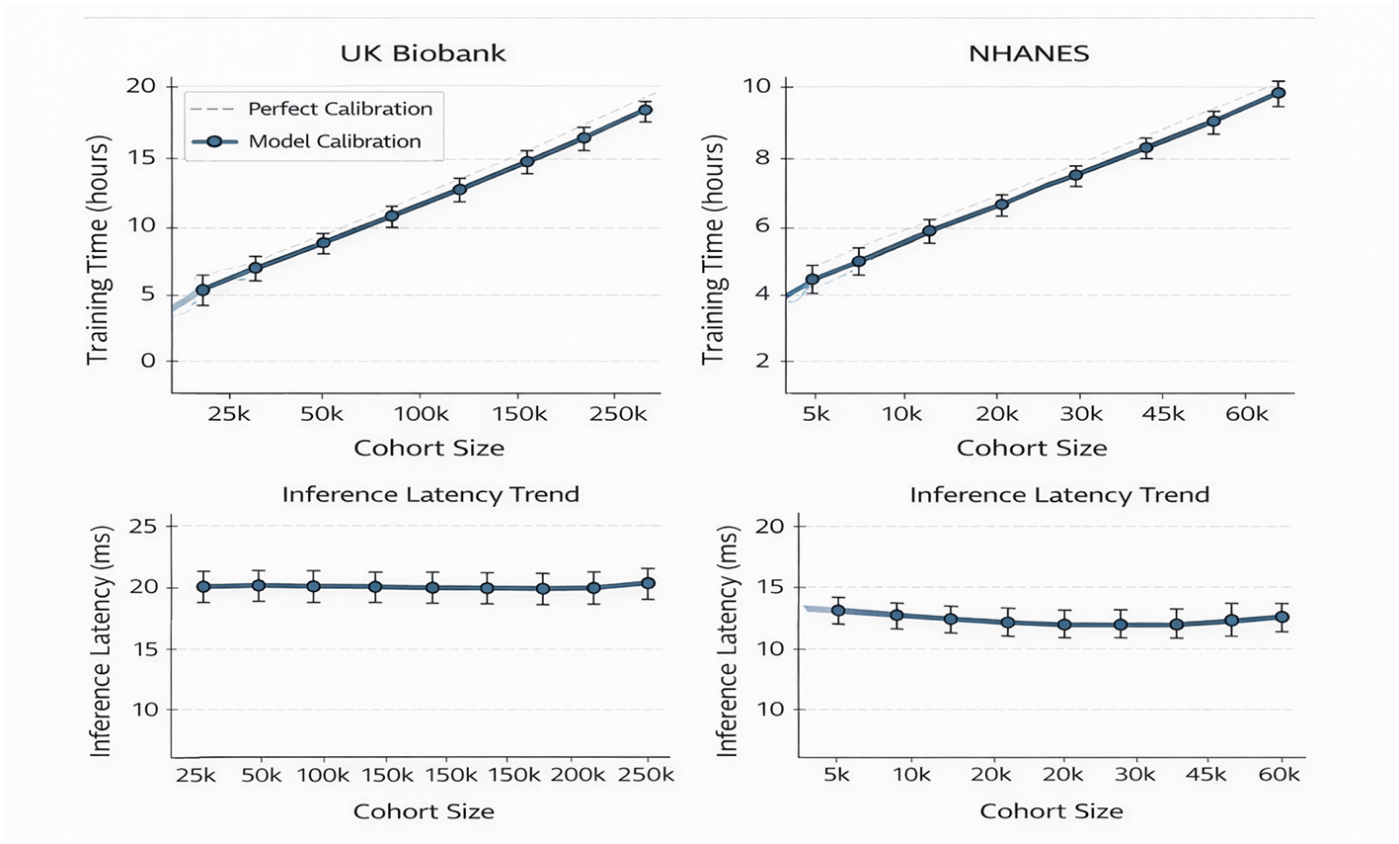
Training time and inference latency as functions of cohort size for UK Biobank and NHANES.

The feasibility of large-scale surveillance of population health is assessed through measurements of scalability and computational efficiency. Training performance and inference time are assessed by varying the number of cohorts and the length of the temporal sequences. The primary evaluation metrics will include total training time, peak memory utilization, and per-person inference latency. The evaluation of scalability metrics will include whether the computational expense of inference grows proportionately with cohort size and whether, when a person is queried, inference across cohorts takes a consistent amount of time regardless of the amount of data used to generate the query. Most importantly, the results showed that, at a near-linear scale with cohort size and with very little variation in latency per person when making inferences with cohorts, this framework will support ongoing, sustained public health monitoring at a large scale. Table 8 presents detailed scalability measurements between the UK Biobank and NHANES, along with the practical ability to use the framework in real-world surveillance pipelines. Figure 8 summarizes the trends regarding training time and inference latency.

**Table 8:** Scalability and computational efficiency analysis on UK Biobank and NHANES datasets.

Cohort size	Training time per epoch	Peak memory	Inference latency per individual	Throughput
UK Biobank				
50k individuals	18.4 ± 0.6 min	6.1 ± 0.2 GB	2.3 ± 0.1 ms	430 ± 12 samples s^−1^
100k individuals	36.9 ± 0.8 min	6.4 ± 0.2 GB	2.4 ± 0.1 ms	421 ± 10 samples s^−1^
250k individuals	92.1 ± 1.3 min	6.9 ± 0.3 GB	2.5 ± 0.1 ms	408 ± 11 samples s^−1^
400k individuals	147.5 ± 2.1 min	7.3 ± 0.3 GB	2.6 ± 0.1 ms	395 ± 13 samples s^−1^
500k individuals	184.2 ± 2.8 min	7.6 ± 0.4 GB	2.6 ± 0.1 ms	389 ± 14 samples s^−1^
NHANES				
20k individuals	7.2 ± 0.3 min	3.4 ± 0.1 GB	1.8 ± 0.1 ms	520 ± 15 samples s^−1^
40k individuals	14.6 ± 0.4 min	3.6 ± 0.1 GB	1.8 ± 0.1 ms	515 ± 14 samples s^−1^
60k individuals	22.1 ± 0.6 min	3.8 ± 0.2 GB	1.9 ± 0.1 ms	508 ± 13 samples s^−1^
80k individuals	29.4 ± 0.7 min	4.0 ± 0.2 GB	1.9 ± 0.1 ms	502 ± 12 samples s^−1^
100k individuals	36.8 ± 0.9 min	4.2 ± 0.2 GB	1.9 ± 0.1 ms	498 ± 11 samples s^−1^

## 5 Discussion

**Figure 9:**
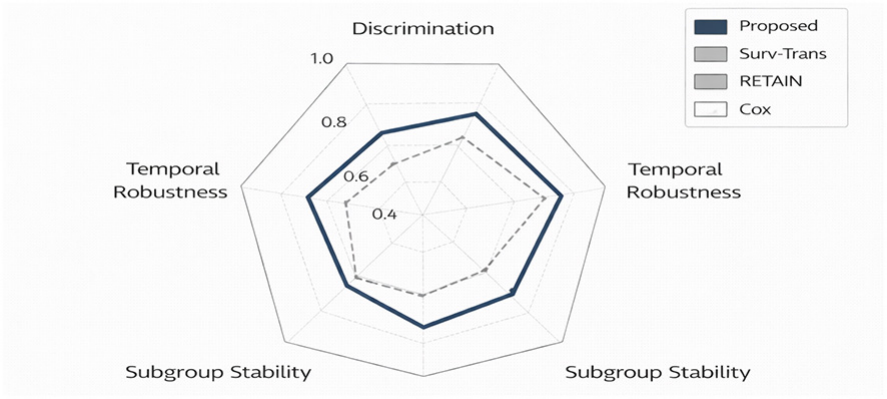
Radar plot summarizing discrimination, temporal robustness, subgroup stability, and scalability across models.

**Figure 10:**
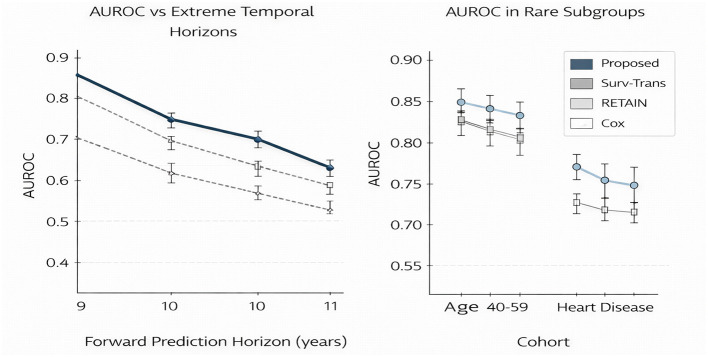
Failure mode analysis highlighting challenging long-horizon predictions and rare population subgroups.

The results of these experiments together show that scalable deep sequence modeling with multi-task supervision is a strong and valid method for surveillance of public health from disparate health care data sources. The improvements demonstrated across various aspects of population discrimination, robustness to temporal horizons, subgroup stability, and throughput processing suggest that co-modeling longitudinal structure and multi-objective surveillance produces a more informative representation of risk than each task's or modality's representation of risk. The consistent improvements observed in both UK Biobank and NHANES suggest that this framework generalizes well across cohort- and survey-based population-sourced data, despite differences in how the data were collected regarding density, temporal granularity, and variable composition. The overall comparative analysis of each data source and its inherent quality for its respective task is presented in Figure 9. Figure 10 analyzes the performance of these measures with respect to extreme temporal horizons and when they reach very rare subgroup representations.

One significant finding is the importance of shared representations of time for learning and capturing population health trends over time and distance. The increasing performance degradation across longer prediction horizons indicates that the model captures significant longitudinal structure rather than only short-term correlation. This is essential for early warning systems because there must be a reliable signal of action taken before the clinical event occurs. Additionally, the stability of model performance across demographic and clinical subgroups indicates that there is only a modest risk of population-level learning objectives overfitting to the majority, given their equitable impact in public health settings.

Operationally, scalability analysis shows that the framework can support multinational deployment without incurring high computational cost. Near-linear growth in training time and consistent prediction latency make it well-suited for ongoing surveillance that requires regular risk updates and fast assessments. High efficiency is especially valuable for sustainable healthcare systems, as it balances resource use and analysis complexity.

The proposed framework shows strong longitudinal surveillance performance when temporally consistent optimization and evaluation settings are applied. However, this study uses a relatively standard hyperparameter optimization framework based on prospective validation combined with a grid search. It is worth noting that the more complex Automated Machine Learning (AutoML) frameworks, Bayesian-optimization-based hyperparameter optimization, Algorithm Selection and Hyperparameter optimization (CASH), and Neural Architecture Search (NAS) methods are omitted here (40). Other recent frameworks combining AutoML with deep Sequential Learning and other frameworks could automatically discover more effective architectures, temporal attention, and large optimization spaces for heterogeneous longitudinal healthcare data. From this perspective, it is possible that more complex AutoML, combined with longitudinal healthcare surveillance modeling frameworks, could benefit the adaptive architecture of large-scale healthcare systems.

Even though all comparative methods were evaluated under the same prospective modeling partitioning and leakage-reduction measures, some recently added longitudinal and surveillance-based benchmarks were conducted using standard or literature-informed configurations instead of full hyperparameter tuning. This was done to maintain methodological consistency and address computational issues across large-scale surveillance experiments. In this context, of all the methods, the most temporally fair evaluation was offered. As the main goal of this research is to analyze the feasibility, robustness, and scalability of temporally consistent population health surveillance, a comparative analysis is appropriate. It is therefore justifiable to explain how the proposed surveillance can be systematized to overcome dominant, longitudinal, and conventional learning methods. Nevertheless, further hyperparameter-tuning studies on specific datasets for some benchmarks may improve the modeling and predictive performance of health surveillance and are highly recommended for the next design to benchmark large-scale healthcare surveillance modeling. Further studies may develop temporally structured healthcare surveillance data to optimize automated machine learning and neural architecture. This includes longitudinal AutoML frameworks such as Auto-Sklong (41) to facilitate model adaptation for diverse population groups.

## 6 Conclusion

This study proposes a multi-scalar deep learning system for population health surveillance as a framework for integrating multiple types of healthcare data into unified time-series or multi-task models. Specifically, the system takes longitudinal observations and constructs aligned surveillance sequences to generate predictions for multiple population-level risk variables. In doing so, this new methodology captures and quantifies temporal patterns of health throughout the lifespan of large populations. Using datasets that are representative both in size and diversity of the populations they represent, the model's data demonstrate increased predictive accuracy, survival concordance, temporal reliability, and subgroup stability. The model's accuracy remained constant as the prediction horizon increased across subpopulations, demonstrating its suitability for equitable public health applications and providing advanced warning of public health events. The findings regarding the model's scalability confirm near-linear computational growth and a predictable inference time, indicating its potential for use at national and international scales within sustainable healthcare systems. Together, these findings demonstrate the importance of developing unified or shared time-scale representations of data to build robust population-health surveillance systems, as well as appropriate methods for incorporating population data into surveillance activities. Future research on the proposed methodology may include additional data modalities used to create predictive models and their effects on public health policy and resource allocation. Overall, the model's methodology represents a technically valid and operationally feasible approach to developing data-driven population health surveillance in real-world settings.

## Data Availability

The original contributions presented in the study are included in the article/supplementary material, further inquiries can be directed to the corresponding author.
